# Protection against brain injury after ischemic stroke by intravenous human amnion epithelial cells in combination with tissue plasminogen activator

**DOI:** 10.3389/fnins.2023.1157236

**Published:** 2023-06-16

**Authors:** Liz J. Barreto-Arce, Hyun Ah Kim, Siow Teng Chan, Rebecca Lim, Grant R. Drummond, Henry Ma, Thanh G. Phan, Christopher G. Sobey, Shenpeng R. Zhang

**Affiliations:** ^1^Department of Microbiology, Anatomy, Physiology, and Pharmacology and Centre for Cardiovascular Biology and Disease Research, School of Agriculture, Biomedicine and Environment, La Trobe University, Bundoora, VIC, Australia; ^2^The Ritchie Centre, Hudson Institute of Medical Research, Clayton, VIC, Australia; ^3^Department of Obstetrics and Gynaecology, Monash University, Clayton, VIC, Australia; ^4^Clinical Trials, Imaging and Informatics (CTI) Division, Stroke and Ageing Research (STARC), Department of Medicine, School of Clinical Sciences at Monash Health, Monash University, Clayton, VIC, Australia

**Keywords:** ischemic stroke, neuroprotection, thrombolytic, stem cells, mouse, inflammation

## Abstract

**Background:**

Thrombolytic agents such as tissue plasminogen activator (tPA) are the only drug class approved to treat ischemic stroke and are usually administered within 4.5 h. However, only ~20% of ischemic stroke patients are eligible to receive the therapy. We previously demonstrated that early intravenous administration of human amnion epithelial cells (hAECs) can limit brain inflammation and infarct growth in experimental stroke. Here, we have tested whether hAECs exert cerebroprotective effects in combination with tPA in mice.

**Methods:**

Male C57Bl/6 mice were subjected to middle cerebral artery occlusion for 60 min followed by reperfusion. Immediately following reperfusion, vehicle (saline, *n* = 31) or tPA (10 mg/kg; *n* = 73) was administered intravenously. After 30 min of reperfusion, tPA-treated mice were injected intravenously with either hAECs (1×10^6^; *n* = 32) or vehicle (2% human serum albumin; *n* = 41). A further 15 sham-operated mice were treated with vehicle (*n* = 7) or tPA + vehicle (*n* = 8). Mice were designated to be euthanised at 3, 6 or 24 h post-stroke (*n* = 21, 31, and 52, respectively), and brains were collected to assess infarct volume, blood–brain barrier (BBB) disruption, intracerebral bleeding and inflammatory cell content.

**Results:**

There was no mortality within 6 h of stroke onset, but a high mortality occurred in tPA + saline-treated mice between 6 h and 24 h post-stroke in comparison to mice treated with tPA + hAECs (61% vs. 27%, *p* = 0.04). No mortality occurred within 24 h of sham surgery in mice treated with tPA + vehicle. We focused on early infarct expansion within 6 h of stroke and found that infarction was ~50% larger in tPA + saline- than in vehicle-treated mice (23 ± 3 mm^3^ vs. 15 ± 2 mm^3^, *p* = 0.02) but not in mice receiving tPA + hAECs (13 ± 2 mm^3^, *p* < 0.01 vs. tPA + saline) in which intracerebral hAECs were detected. Similar to the profiles of infarct expansion, BBB disruption and intracerebral bleeding in tPA + saline-treated mice at 6 h was 50–60% greater than in vehicle-treated controls (2.6 ± 0.5 vs. 1.6 ± 0.2, *p* = 0.05) but not after tPA + hAECs treatment (1.7 ± 0.2, *p* = 0.10 vs. tPA + saline). No differences in inflammatory cell content were detected between treatment groups.

**Conclusion:**

When administered following tPA in acute stroke, hAECs improve safety and attenuate infarct growth in association with less BBB disruption and lower 24 h mortality.

## Introduction

Ischemic stroke is the leading cause of death and disability globally (Campbell et al., 2019; Krause et al., 2019). It occurs when a thrombus or embolus obstructs blood flow in a cerebral artery which interrupts oxygen and glucose supply to a region of the brain (Campbell et al., 2019; Zhang et al., 2021). Currently, thrombolytics such as tissue plasminogen activator (tPA; i.e. Alteplase and Tenecteplase) are the only drug class approved for acute ischemic stroke (Gravanis and Tsirka, 2008; Campbell et al., 2019; Ma et al., 2019). This therapy is typically administered within 4.5 h of stroke onset after imaging has confirmed thrombosis and a salvageable penumbra, but this narrow time window precludes ~80% of stroke patients from eligibility to receive tPA (Adibhatla and Hatcher, 2008; Gravanis and Tsirka, 2008). Furthermore, tPA therapy may result in incomplete lysis of clots, and has other drawbacks including damage to the basal lamina of cerebral blood vessels and increased risk for hemorrhagic transformation, edema and neurotoxicity (Adibhatla and Hatcher, 2008; Vivien, 2017). It therefore remains important to identify additional treatments that could benefit stroke patients when administered alone or in combination with tPA.

Cell-based therapies have gained interest for treating acute stroke because they have immunomodulatory properties which can exert potent anti-inflammatory actions to limit inflammation-driven infarct expansion that occurs secondary to ischemic brain injury (Zhang et al., 2020, 2021; Zhang and Lai, 2020). For example, human amniotic epithelial cells (hAECs), which can be harvested from term placentas after delivery, have low immunogenicity (and thus do not require immunosuppressant therapy to prevent rejection after transplantation) and low tumorigenicity (Miki et al., 2005) and can suppress proinflammatory cytokines, regulate macrophage recruitment and secrete neurotrophic factors (Castillo-Melendez et al., 2013). We demonstrated that early intravenous administration of hAECs can limit brain inflammation and infarct growth in several models of experimental stroke (Evans et al., 2018). Those findings led to a Phase 1 clinical trial of hAECs in acute stroke patients that was recently completed (Phan et al., 2023). Here, we have tested whether hAECs can protect against brain injury in acute ischemic stroke when administered intravenously following tPA in mice.

## Methods

### Animals

This study was approved by the La Trobe University Animal Ethics Committee (AEC 17–79) and performed according to the National Health and Medical Research Council of Australia code for the care and use of animals for scientific purposes. A total of 179 male C57BL/6 mice (aged 8–12 weeks) were studied. Sixty mice were excluded from the study because they: (i) died during the surgical procedure (*n* = 13) or within 5 min after bolus injection of cells due to probable pulmonary embolism (*n* = 15); (ii) experienced subarachnoid hemorrhage during intracerebral artery filament insertion (*n* = 18); or (iii) were euthanised due to not meeting inclusion criteria, i.e., <65% reduction in regional cerebral blood flow (rCBF) during stroke or < 50% recovery of rCBF within 5 min of reperfusion (*n* = 14). Animals were housed on a 12 h dark/light cycle and fed *ad libitum*.

### Middle cerebral artery occlusion (MCAO) model of stroke

MCAO surgery was performed as described (Kim et al., 2014; Evans et al., 2018). Briefly, mice were anesthetised (ketamine 100 mg/kg and xylazine 10 mg/kg intraperitoneally; i.p.). Local anaesthetic (2 mg/kg bupivacaine; subcutaneously, s.c.) was injected in the incision area of the neck and rectal temperature was monitored and maintained at 37 ± 0.5°C throughout the surgery. The right common and external carotid arteries (CCA and ECA, respectively) were exposed. The external carotid artery was ligated and a vessel stump was made, thus permanently closing that artery after filament retraction. A small opening was made on the stump and a 6.0 silicone-coated monofilament was inserted through the opening and gently advanced into the internal carotid artery (ICA) to obstruct the origin of the right middle cerebral artery (MCA). The occlusion was maintained for 60 min, followed by filament withdrawal to allow reperfusion. Both successful occlusion (>65% reduction in cerebral blood flow; CBF) and reperfusion (>50% CBF to pre-ischemic level) were corroborated by transcranial Laser-Doppler flowmetry (Perimed). Neck and head incisions were then closed. All animals received 1 mL of sterile saline s.c. for hydration and returned to their cages after regaining consciousness.

### hAEC preparation for injection

The collection and use of placentas was approved by the Monash Health Human Research Ethics Committee (12223B). All donors gave written, informed consent for the collection of their placenta. Placentas were collected from healthy donors undergoing elective caesarean section for the delivery of a singleton birth. A total of 3 placentas were used in this study, with cells from only one donor administered to each mouse. Briefly, immediately after delivery, the amniotic membrane was stripped from the chorion membrane of the placenta. The amniotic membrane was then repeatedly washed in Hanks’ Balanced Salt Solution followed by digestion in trypsin solution. Isolated cells were filtered, pooled and assessed for purity (Murphy et al., 2010). Primary hAEC isolates were gated positive for the epithelial cell marker, epithelial cell adhesion molecule (EpCAM), and negative for mesenchymal stromal markers CD90 and CD105 (Evans et al., 2018). Cells were cryopreserved in liquid nitrogen until use. Cells were thawed and washed with 2% human serum albumin (HSA) in saline to remove any excess dimethylsulfoxide (DMSO). The viability of cells post-thaw was assessed by trypan blue, and a cellular suspension of 5×10^6^ cells/ml with viability ≥80% was used. It is possible that cells from different donors vary in ‘quality’ in their therapeutic potential, however we are not aware of, and have not previously been able to identify any donor-dependent effects on hAEC efficacy following experimental stroke.

### Experimental design

Mice that underwent MCAO surgery were randomised into three treatment groups. Mice were injected intravenously (i.v.) with either: (1) vehicle only (200 μL of 2% HSA in saline) after 30 min of reperfusion, (2) tPA (Alteplase, 10 mg/kg; Boehringer Ingelheim) at the start of reperfusion and then vehicle after 30 min, or (3) tPA at the start of reperfusion and then hAECs (1×10^6^ cells in 200 μL of 2% HSA in saline) after 30 min. A further 15 mice were sham-operated and then treated with vehicle (*n* = 7) or tPA + vehicle (*n* = 8) after 30 min. For sham surgery, we made a neck incision and exposed the CCA, ECA, ICA and bifurcation. No further surgical procedure or vessel ligation was made. Mice were euthanised according to designated endpoint times at 3 h (*n* = 21), 6 h (*n* = 31) or 24 h (*n* = 50) post-stroke. Animals euthanised at 24 h were assessed for functional deficits in two tests (see below). Brains were collected to determine infarct volume and assess markers of injury and inflammation.

### Functional assessment

At 24 h after MCAO, functional assessments were performed, as described (Evans et al., 2018). Clinical scoring was performed using a 6-point scoring system: 0 = normal motor function, 1 = flexion of torso and contralateral forelimb when mouse is lifted by the tail, 2 = circling to the contralateral side when mouse held by the tail on a flat surface but normal posture at rest, 3 = leaning to the contralateral side at rest, 4 = no spontaneous motor activity, 5 = death prior scheduled endpoint. Latency to fall in the hanging grip test was used to assess grip strength. In this test, the time for which a mouse could remain suspended on a wire 60 cm above a soft surface was recorded, and the average time of 3 trials with 2 min rest in between was calculated. The maximum time per trial was 180 s. All behavioural tests and further analyses were performed by an operator blinded to the group allocation of the animals.

### Infarct analysis

At the endpoint of experiments (3 h, 6 h or 24 h post-stroke), mice were euthanised by inhalation of CO_2_ and decapitation, and the brain was immediately removed, frozen in liquid nitrogen and stored at −80°C until sectioning. Coronal sections (30 μm) separated by ~840 μm were obtained and stained with thionin (0.1%) to delineate the infarct (Jackman et al., 2009; Evans et al., 2018). Briefly, images of sections were captured, and infarct area was measured manually using ImageJ software (NIH) in at least six 30 μm sections per brain that were spaced 840 μm apart by an experienced researcher blinded to the treatment group. Infarct volume for each brain was then estimated based on these six measurements. Infarct volume was measured by applying the formula: corrected infarct volume = [left hemisphere area – (right hemisphere area – right hemisphere infarct area) x (thickness of section + distance between sections)] (Jackman et al., 2009; Kim et al., 2014).

### Immunohistochemical staining with chromogenic detection using 3,3′-diaminobenzidine (DAB) staining

DAB staining was performed to identify neutrophils. Briefly, 4% paraformaldehyde (PFA) fixed coronal brain sections were blocked with peroxidase blocking solution (REALTM EnVisionTM Detection System, K5007, DAKO) for 10 min. After washing with phosphate-buffered saline (PBS), sections were blocked with 10% normal goat serum (NGS) for 40 min. Sections were rinsed and incubated with rabbit polyclonal anti-myeloperoxidase (MPO; 1:100; ab9535, Abcam) antibody overnight at 4°C. Following washing, sections were incubated with peroxidase-labelled polymer conjugated goat anti-rabbit immunoglobulin (DAKO) for 2 h at room temperature. Further, sections were washed and incubated with DAB reagent (DAKO) for 5 min in the dark, rinsed in distilled water and then counterstained with haematoxylin (1:4) for 30 s. Sections were then rinsed in Scott’s tap water for 1 min and dehydrated through an ascending ethanol series, immersed in xylene for 1 min and coverslipped with dibutylphthalate polystyrene xylene (DPX, Sigma) mounting medium. MPO^+^ neutrophils were counted by a researcher blinded to the sample identity.

### Immunofluorescence

Coronal sections were stained to identify microglia/macrophages (ionised calcium-binding adapter molecule 1, Iba-1^+^), macrophages (F4/80^+^) and oxidative injury (3-nitrotyrosine; 3NT^+^). Human leukocyte antigen-G (HLA-G) antibody was used to detect the presence of hAECs in the brain, whilst IgG and matrix metalloproteinase-9 (MMP-9) were stained to assess for blood–brain barrier (BBB) dysfunction. Frozen brain sections (10 μm) were fixed with ice-cold 4% PFA in 0.01 M PBS for 15 min. Then, sections were blocked with 10% NGS + 0.3% Triton X in PBS for 1 h, followed by overnight incubation with the following primary antibodies: rat monoclonal anti-F4/80 (1:50; ab6640, Abcam), mouse monoclonal anti-3-nitrotyrosine (anti-3NT; 1:50; ab61392, Abcam), rabbit polyclonal anti-ionised calcium-binding adapter molecule 1 (anti- Iba-1, 1:200; #17198, Cells Signalling Technology), mouse monoclonal anti-HLA-G (1:500; ab52455; Abcam) and rabbit anti- MMP9 (1:70, ab38898, Abcam). Double-labelled immunostaining (F4/80 + 3NT) and HLA-G staining were performed as described (Evans et al., 2018; Kim et al., 2020). After washing, sections stained with a mouse antibody were processed according to the manufacturer’s protocol using a Mouse-on-Mouse Kit™ (FMK-2201, Vector Laboratories) and the remaining sections were incubated with a respective secondary antibody (goat anti-rabbit IgG Alexa 594 or goat anti-rat IgG Alexa 594, 1:500, ThermoFisher) for 2 h. Finally, sections were cover-slipped using Vectorshield mounting medium with 4,6-diamino-2-phenylindole (DAPI Vector Laboratories).

For IgG staining, 4% PFA-fixed slides were blocked with NGS and incubated with Alexa 594 goat anti-mouse antibody (1:200, A-11032, Invitrogen) for 2 h. Then, sections were washed and coverslipped with Vectorshield mounting medium with DAPI. Brain sections were evaluated using the filters Cy3 (for F4/80, IgG, Iba-1 and MMP-9) or FITC (for 3NT and HLA-G). Cells positive for F4/80, 3NT and/or HLA-G were counted in the ischemic hemisphere and also the contralateral hemisphere in the case of MMP-9. IgG and Iba-1 staining was assessed using ImageJ software. IgG was evaluated by intensity, and Iba-1 positive cells were counted per hemisphere section. Cells were quantified and images were captured using a light microscope (BX53, Olympus™) with a fluorescence lamp (X-Cite™ 120Q) attached, and with 20x or 40x objective lenses (UplanFL N, Olympus™). Images were taken in the ipsilateral hemisphere (infarct, peri infarct and cortical regions) and corresponding locations in the contralateral hemisphere were included in the analyses. All sections were imaged on the same day using the same settings and analysed by a researcher blinded to the sample identity.

### Statistical analysis

D’Agostino & Pearson or Kolmogorov–Smirnov normality tests were performed to assess distribution profiles of each variable in each data set. Normally distributed data were analysed using one-way ANOVA tests with Dunnett’s *post hoc* test for multiple comparisons. Non-normally distributed or categorial data were analysed using Kruskal-Wallis tests. Mortality rate at 24 h was compared using the Chi-square test. Values are expressed as mean ± standard error of the mean (SEM). All statistical analysis were performed using GraphPad Prism version 8.02 (GraphPad Software, CA, United States). Statistical significance was accepted when *p* < 0.05.

## Results

### Clinical score and functional deficits at 24 h after stroke

Our main goal was to test whether hAECs can protect against brain injury in acute ischemic stroke *in vivo* in the presence of tPA. In the initial cohort of mice designated for functional assessment and euthanasia at 24 h, the median clinical score was higher in those treated with tPA than in vehicle-treated mice subjected to stroke (median score = 5 vs. 3, respectively; Supplementary Figure S1A). This worsening of the 24 h post-stroke median clinical score by tPA alone was mainly due to a higher mortality (61%; 14/23) than in vehicle-treated controls (29%; 4/14, *p* = 0.057 vs. tPA + vehicle, Chi-square test; Supplementary Figure S1A). Importantly, administration of hAECs in combination with tPA resulted in a lower mortality (27%; 4/15; *p* = 0.039 vs. tPA + vehicle, Chi-square test), indicating that hAECs exerted protective actions against severe adverse effects of tPA. Furthermore, we confirmed that the greater mortality in tPA-treated mice was related to ischemic stroke pathology because no mortality occurred over 24 h in an additional 8 sham-operated mice treated with tPA + vehicle (Supplementary Figure S2).

As key endpoint measures (hanging grip time; fully established infarct volume) must be assessed in mice that survive for 24 h in this model of stroke (i.e., in those mice tending to have the mildest pathology), such different mortality rates between the treatment groups prevented a true and unbiased assessment of hAECs efficacy on these endpoint measures. Nevertheless, such data collected from these mice are shown in Supplementary Figures S1B,C, indicating similar hanging grip performances and infarct volumes amongst mice that survived to 24 h.

### Infarct development

**Figure 1:**
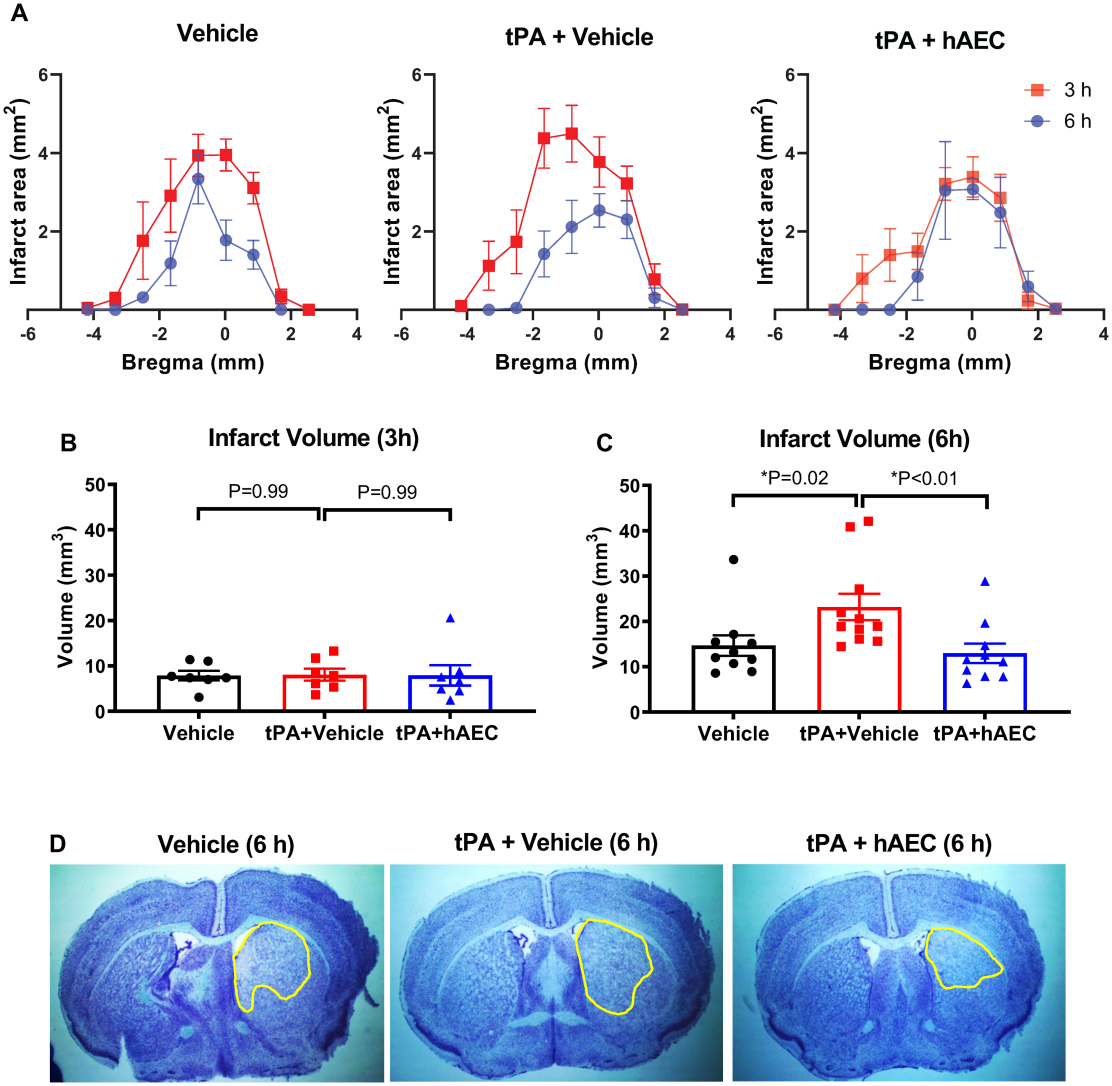
Infarct distribution and volume. **(A)** Comparison of infarct distribution based on bregma location (*n* = 6–11 per group). Infarct size at **(B)** 3 h (*n* = 7 per group) and **(C)** 6 h (*n* = 10–11 per group) post-stroke. Representative images of delineated infarcts at 6 h are shown in **(D)**. Data are presented as mean ± SEM, *p < 0.05 compared with tPA + vehicle, **(B)** one-way ANOVA test with Dunnett’s *post hoc* test, **(C)** Kruskal-Wallis test with Dunn’s *post hoc* test.

As the unexpected tPA-related effects on overnight survival precluded the testing of our hypothesis within the original protocol, we instead tested if early cerebroprotective effects of hAECs on infarct growth might be apparent before the period of mortality associated with tPA treatment (i.e., ≤6 h). Thus, in additional groups of mice treated with vehicle, tPA + vehicle or tPA + hAECs, infarct distribution and size were assessed at 3 h (all groups were *n* = 7) or 6 h (*n* = 10, 11 and 10, respectively) after MCAO. No deaths occurred in any of these animals before the scheduled time for euthanasia (i.e., 3 h or 6 h). Our findings revealed that substantial growth of the infarct core occurred between 3 h and 6 h after MCAO in mice treated with vehicle alone or tPA + vehicle, but not tPA + hAECs (Figures 1A-D). Furthermore, whereas there was no effect of tPA and/or hAECs on infarct volume at 3 h (Figure 1B), by 6 h it was apparent that infarct growth was augmented by ~50% by tPA + vehicle, but not by tPA + hAECs, in comparison to vehicle only (Figures 1C,D). Average increase in infarct volume from 3 h to 6 h was: vehicle: 7.9 mm^3^ to 14.7 mm^3^ = 86%; tPA + vehicle: 8.1 mm^3^ to 23.2 mm^3^ = 186%; tPA + hAECs: 7.9 mm^3^ to 13.0 mm^3^ = 65%. Thus, infarct growth rate was 2-to 3-fold greater in mice treated with tPA + vehicle than in the other groups.

### hAEC localisation

**Figure 2:**
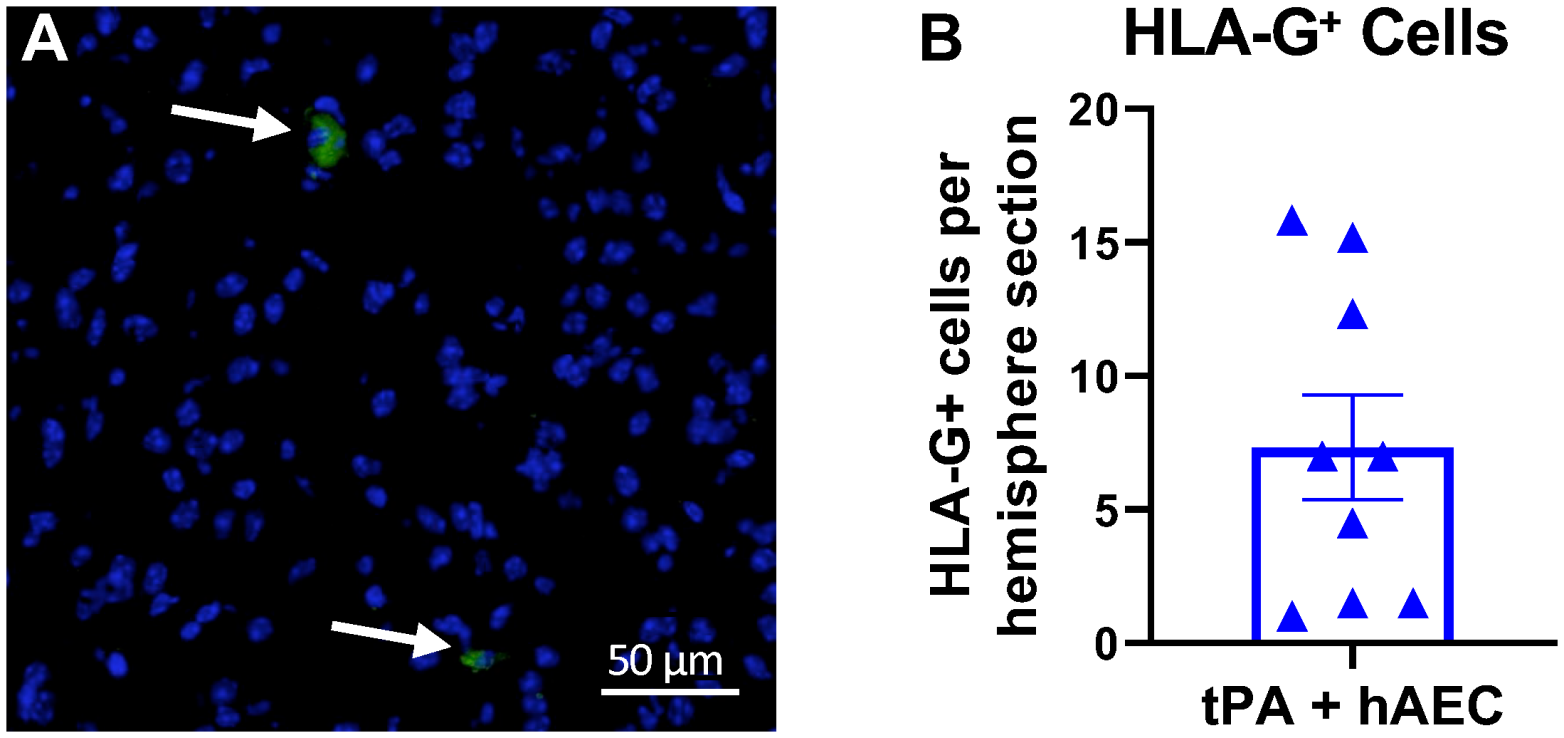
Human amnion epithelial cells reach the ipsilateral hemisphere at 6 h post-stroke. **(A)** Immunofluorescence staining for human HLA-G (green, arrows) revealed the presence of hAECs in the ischemic hemisphere. Nuclei are identified by DAPI counterstain (blue). **(B)** Number of HLA-G^+^ cells in the ipsilateral hemisphere (*n* = 9 mice; average of 6 sections per mouse). Objective: 40X.

Brain sections stained for HLA-G antibody revealed that hAECs injected i.v. at 1.5 h post-stroke were present in the ischemic hemisphere within 6 h (*n* = 9 mice, Figure 2). In each brain we examined six 10 μm sections, 420 μm apart, and found that the average number of hAECs in the ischemic hemisphere was 7.3 per section (Figure 2B). From these data and measurement of infarct length, we estimated that ~0.4% of hAECs administered (i.e., ~4,000 hAECs) had localised within in the ischemic hemisphere by 6 h post-stroke.

### Intracerebral hemorrhage

**Figure 3:**
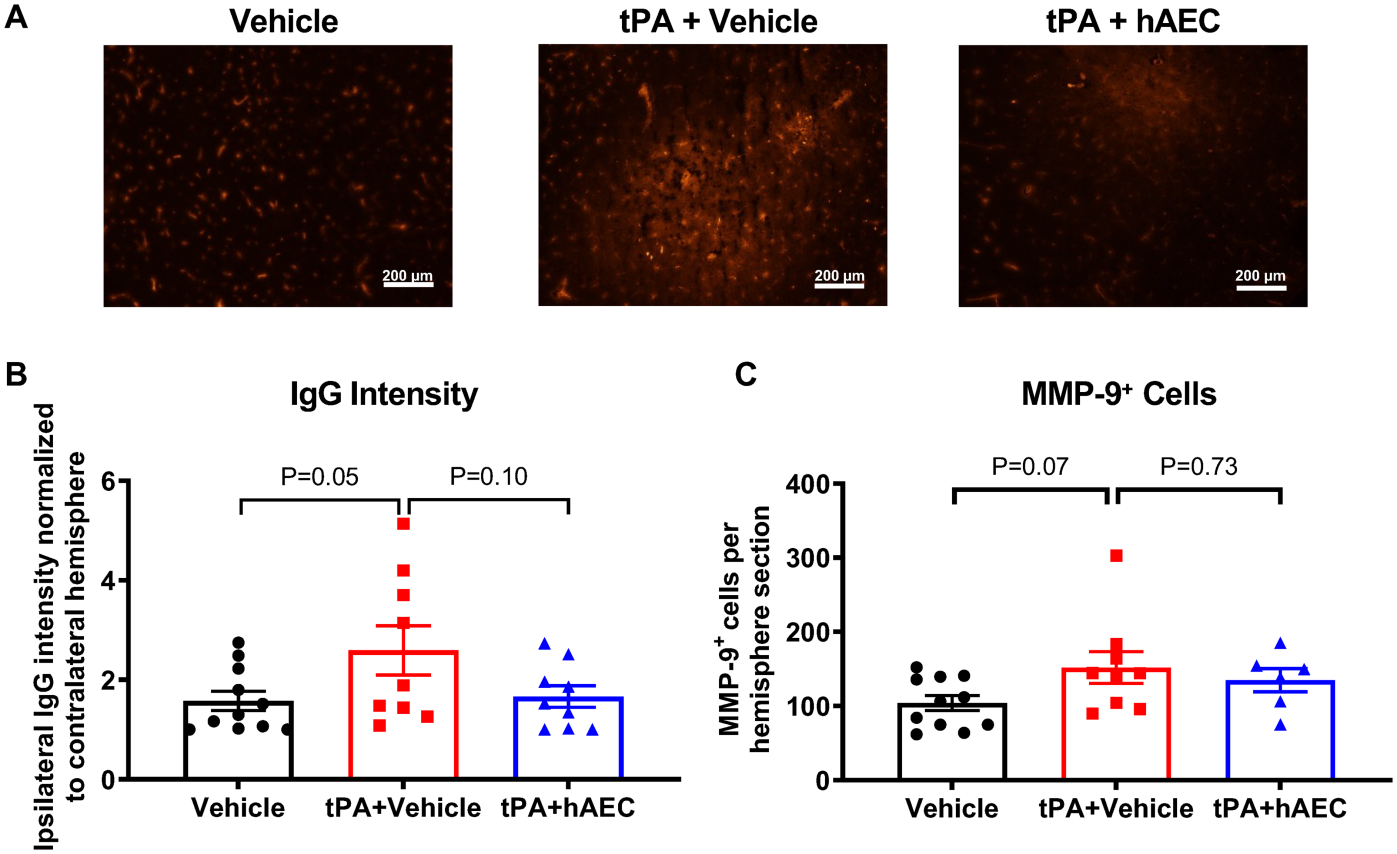
Blood–brain barrier disruption at 6 h post-stroke. **(A)** Representative images of brain sections showing IgG leakage (anti-mouse antibody) into the ipsilateral hemisphere after stroke. **(B)** Quantification of extravasation of IgG in terms of intensity (*n* = 9–11 per group). **(C)** Number of MMP-9^+^ cells in the ipsilateral hemisphere of the brain (*n* = 6–11 per group). Data are expressed as mean ± SEM. One-way ANOVA test with Dunnett’s *post hoc* test. Scale bar = 200 μm. Objective: 10X.

Intracerebral hemorrhage was visible in unstained brains from several mice, and perhaps especially in those treated with tPA + vehicle (see Supplementary Figures S3A-C for representative images). To estimate the extent to which the BBB was injured, leading to bleeding into the brain parenchyma after stroke, brain sections were systematically stained for IgG. Immunofluorescence intensity revealed that BBB dysfunction in the ischemic hemisphere was augmented in mice treated with tPA + vehicle versus vehicle only (Figures 3A,B; *p* = 0.05); but was not augmented in tPA + hAEC-treated mice (Figures 3A,B; *p* = 0.97 vs. vehicle only). Amongst all brains examined at 6 h post-stroke there was a significant positive correlation between infarct volume and IgG intensity, consistent with ischemic brain injury occurring in association with BBB disruption (Supplementary Figure S3D). Furthermore, in brains from tPA + hAEC-treated mice, there tended to be a negative correlation between IgG intensity and HLA-G^+^ cells, consistent with the possibility that hAECs inhibited BBB breakdown (Supplementary Figure S3E). MMP-9 was also assessed because its expression is related to BBB disruption, and we noted that even by 6 h following stroke there appeared to be a trend for more MMP-9-positive cells in the ipsilateral hemisphere of tPA + vehicle-treated mice than in mice treated with vehicle only (Figure 3C). However, there was no effect of hAEC treatment on the number of MMP-9-positive cells (Figure 3C).

### Inflammatory cells

**Figure 4:**
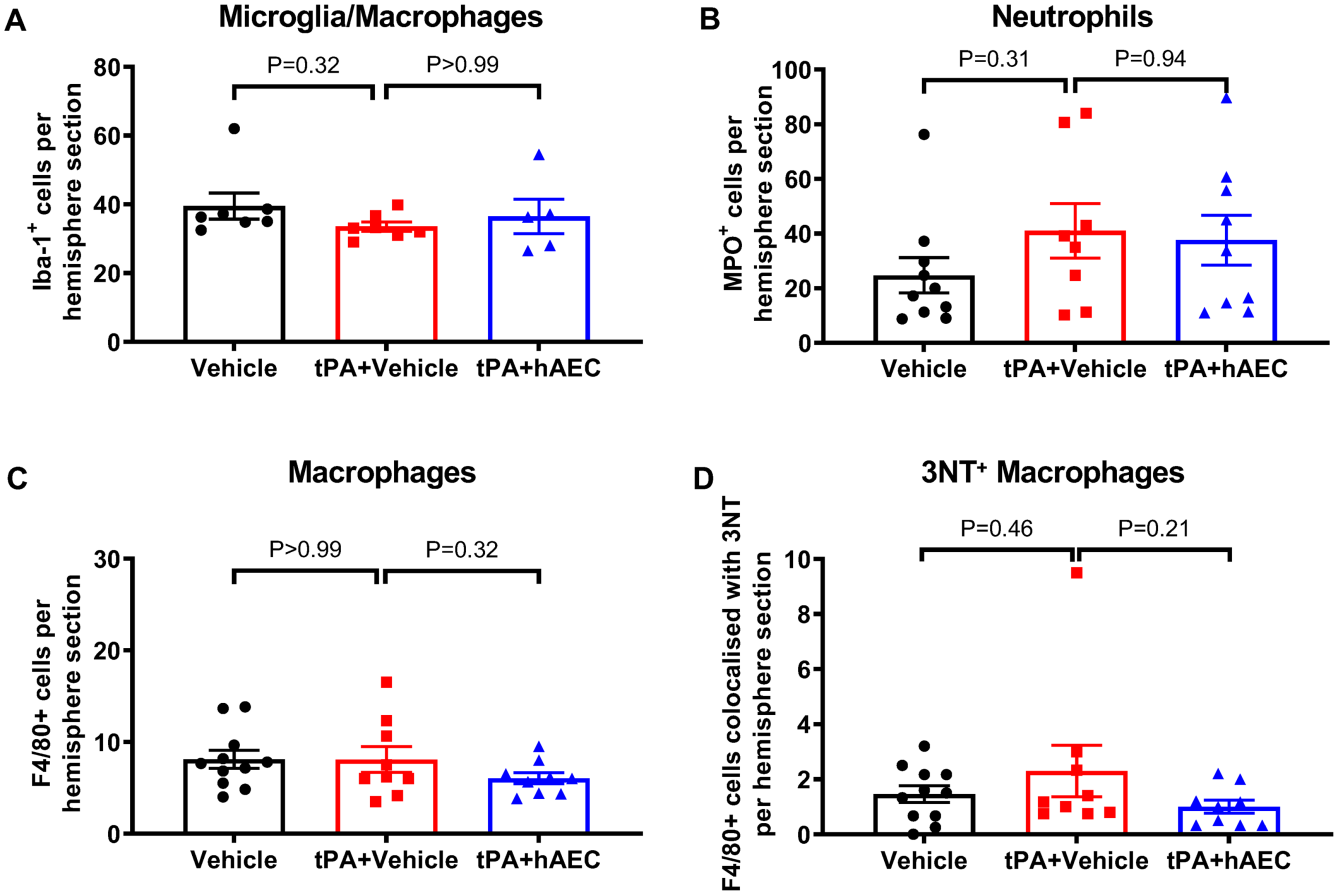
Immune cell numbers in the ipsilateral hemisphere at 6 h post-stroke. Quantification of **(A)** microglia/macrophages (Iba-1^+^ cells, *n* = 5–7 per group), **(B)** neutrophils (MPO^+^ cells, *n* = 8–10 per group), **(C)** macrophages (F4/80^+^ cells, *n* = 9–11 per group), and **(D)** 3-nitrotyrosine (3NT)^+^ F4/80^+^ cells (*n* = 9–11 per group) in the ipsilateral hemisphere at 6 h post-stroke. **(A,C,D)** were evaluated by immunofluorescence, whilst **(B)** was assessed by immunohistochemistry. Data are expressed as mean ± SEM. **(A)** Kruskal-Wallis test with Dunn’s *post hoc* test. **(B-D)** One-way ANOVA test with Dunnett’s *post hoc* test.

To further explore potential mechanisms of hAEC-induced protection against infarct expansion in the presence of tPA, we assessed levels of inflammatory cells that may be associated with ischemic lesion formation in the mouse model of MCAO. We found no difference between groups in the number of microglia/macrophages (Iba^+^ cells; Figure 4A), neutrophils (MPO^+^ cells; Figure 4B) or macrophages (F4/80^+^ cells; Figure 4C) in the ischemic hemisphere. However, we noted a trend for fewer pro-inflammatory macrophages (F4/80^+^ 3NT^+^ cells) in the ischemic hemisphere of mice treated with tPA + hAECs compared with tPA + vehicle (Figure 4D, *p* = 0.21), consistent with a possible anti-inflammatory effect of hAECs. Representative images are shown in Supplementary Figure S4.

## Discussion

This study has examined whether post-stroke intravenous administration of hAECs can limit brain injury in mice when given in combination with the thrombolytic agent, tPA. We found that hAEC therapy reduced 24 h mortality, as well as early infarct expansion, BBB disruption and intracerebral bleeding in tPA-treated mice. The findings support the broadening of clinical use of hAEC therapy in patients that have received thrombolytic therapy.

Thrombolytic agents such as tPA comprise the only drug class approved for acute ischemic stroke (Gravanis and Tsirka, 2008; Campbell et al., 2019). This therapy is usually administered within 4.5 h of stroke onset (Gravanis and Tsirka, 2008; Campbell et al., 2019) but can be extended to at least 9 h when imaging has confirmed the presence of salvageable tissue (Ma et al., 2019). Although tPA can promote cerebral reperfusion it also increases the risk of intracerebral hemorrhage (Gravanis and Tsirka, 2008; Vivien, 2017; Boese et al., 2020) and may negatively affect neuronal viability (Wang et al., 1998) and exert direct neurotoxic effects (Vivien, 2017; Zhu et al., 2019; Yepes et al., 2021). Overall, thrombolytic therapy benefits only ~20–25% of stroke patients (Vanacker et al., 2016; Stroke Foundation, 2017). Besides thrombolytic and surgical interventions to promote reperfusion, there is an interest in exploiting other targets to modulate detrimental cellular mechanisms that exacerbate post-ischemic brain injury. For example, in an attempt to target inflammation-driven infarct growth, we previously demonstrated that early intravenous administration of hAECs can limit brain inflammation and infarct growth in several experimental stroke models (Evans et al., 2018). Those findings led to a Phase 1 clinical trial of hAECs in acute stroke patients that was recently completed (Phan et al., 2023). However, it is not known whether hAECs are safe and effective when given in combination with tPA following stroke. To address this knowledge gap, here we assessed whether intravenous administration of hAECs can exert cerebroprotective effects in acute ischemic stroke following tPA treatment. Thus, given that there could be a potential interaction, we administered hAECs in combination with tPA to ensure that this cell therapy is compatible with the prior administration of tPA. As this question is not related to any potential neuroprotective properties of tPA nor its thrombolytic capacity, it could be addressed in the filament model of MCAO stroke, which lacks a clot.

In the initial cohort of mice designated for functional assessment and euthanasia at 24 h, the median clinical score was higher in mice treated with tPA + vehicle than in vehicle-treated mice subjected to stroke. This worsening of the 24 h post-stroke median clinical score by tPA + vehicle was mainly due to higher mortality than in vehicle-treated controls. However, when tPA was administered in combination with hAECs, there was no such tPA-induced increase in mortality or clinical score, suggesting that hAECs exerted protective actions against severe adverse effects of tPA. Furthermore, we confirmed that the greater mortality in tPA + vehicle-treated mice was related to ischemic stroke pathology because no mortality occurred in an additional 8 sham-operated mice treated with tPA + vehicle. Thus, our findings firstly suggest that hAECs could reduce mortality, possibly secondary to intracerebral hemorrhage (see below), associated with tPA administration after stroke.

The unexpected mortality rate in tPA + vehicle-treated mice occurred overnight between 6 to 18 h post-stroke, which precluded a proper assessment of hAECs on 24 h infarct volume in the presence of tPA. We presume that tPA-related BBB dysfunction and intracerebral bleeding in our filament model of ischemic stroke contributed to this effect. Therefore, we instead tested if the cerebroprotective effects of hAECs on early infarct growth were apparent within 6 h of cerebral ischemia (i.e., within 4.5 h after hAEC administration). Thus, in additional groups of mice treated with vehicle, tPA + vehicle or tPA + hAECs, infarct size was assessed at 3 h or 6 h after MCAO. Our findings provide evidence that hAECs reduced infarct growth between 3 h and 6 h post-stroke. Furthermore, we noted that by 6 h infarct growth was augmented by tPA alone, but not by tPA + hAECs, in comparison to vehicle only.

It is noteworthy that tPA is conventionally administered at a higher dose in rodents than in humans because the fibrinolytic system is reportedly 10-fold less sensitive in rodents (Korninger and Collen, 1981; Kilic et al., 2001). Indeed, in the present study, we used the standard intravenous dose used in rodents (10 mg/kg). However, the unexpectedly high 24-h mortality associated with tPA administration post-stroke caused us to consider the possibility that 10 mg/kg tPA was excessive in this filament-induced cerebral artery occlusion model, which does not involve clot formation. However, in pilot experiments using a lower tPA dose (0.9 mg/kg, used clinically) we found similar 24-h post-stroke mortality (4/5; data not shown). This high mortality could be related to the neurotoxicity of tPA (Harston et al., 2010) and/or increased BBB permeability.

We evaluated BBB disruption and intracerebral bleeding by immunostaining for IgG (Kassner and Merali, 2015). We found that compared with mice treated with vehicle only, there was evidence of greater BBB dysfunction in the ischemic hemispheres of mice treated with tPA + vehicle by 6 h, consistent with the known effects of tPA to promote hemorrhagic transformation, which limits its clinical utility (Kim, 2019). Interestingly, we found that hAECs administered in combination with tPA appeared to reduce the magnitude of this intracerebral bleeding, analogous to reports from other studies of post-stroke cell therapy (Liu et al., 2012; Nakazaki et al., 2017; Boese et al., 2020). Intravenous tPA is reported to increase BBB permeability in rats in association with neurotoxicity, brain damage and mortality in a manner that contributes to its brief therapeutic window (Fanne et al., 2010). We speculate that tPA-induced intracerebral hemorrhage contributed to the augmented early infarct growth and that the protective effects against hemorrhage by hAECs contributed to their protective effects against infarct growth. Indeed, there was a positive correlation between infarct volume and IgG intensity, consistent with ischemic brain injury occurring in association with BBB disruption. Furthermore, we detected the presence of hAECs in the ischemic hemisphere by 6 h in numbers that tended to be associated with less bleeding, consistent with the cells exerting direct protective actions against post-ischemic BBB breakdown and brain injury. It has similarly been reported that such an effect of mesenchymal or neural stem cells is associated with reduced IgG intensity (Cheng et al., 2018; Boese et al., 2020). However, whilst there was a trend for more MMP-9-positive cells in the ischemic hemisphere of mice treated with tPA + vehicle, there was no apparent effect of hAECs on this.

Ischemic stroke pathology may involve activation and proliferation of resident microglia/macrophages and infiltration of circulating inflammatory cells into the brain to cause further injury and death of the penumbral tissue (Zhang et al., 2021). We previously found that, in the absence of tPA, cerebroprotective effects of intravenous hAECs were associated with reduced infiltration of neutrophils and macrophages in the ischemic hemisphere by 24 h (Evans et al., 2018). In the present study, we found little or no evidence that reduced numbers of immune cells were associated with cerebroprotection by hAECs within 6 h. Specifically, whilst there was a trend for fewer macrophages (both total and especially with a pro-inflammatory phenotype) there was no statistical difference in numbers of neutrophils or microglia/macrophages in the ischemic hemisphere at 6 h. These data imply that hAECs did not provide early cerebroprotection via significant attenuation of inflammatory cell activity.

Although a limitation of the study is that only acute and subacute time points were included, our goal was specifically to evaluate hAECs in the context of tPA therapy, which itself is only clinically relevant for this post-stroke period given its narrow therapeutic time window and short half-life. Thus, we were still able to address our specific question whilst avoiding confounding of results due to tPA-induced mortality. Furthermore, our previous studies have comprehensively examined histological and functional outcomes of hAEC therapy in stroke models both acutely and long-term (Evans et al., 2018), according to the stroke therapy academic industry roundtable preclinical recommendations (Fisher et al., 2009).

It is noteworthy that a number of mice were excluded from the study because they died within 5 min after bolus injection of cells due to probable pulmonary embolism. We have observed this phenomenon in preclinical studies, in which the risk of losing animals due to pulmonary embolism increases with the dose of cells infused. Intravenous infusion is commonly used in both preclinical and clinical trials of cell-based therapies where it is well known that infused cells may be rapidly trapped in the lung, followed by their relocation to the internal organs (Cui et al., 2015). Although the diameter of hAECs is only 8–15 μm (Broughton et al., 2013), they may potentially form a cluster and block pulmonary capillaries as they are the first capillaries reached after intravenous injection. Our broader experience has revealed that such events can be greatly limited by performing slow infusions, and hAECs are infused in humans over 1 h (Phan et al., 2023). However, this is extremely difficult in mice in which the cells must be infused in a relatively small volume (0.2 mL). We acknowledge that transient hypoxia could therefore be a risk associated with infusion of hAECs into patients, and the risk and severity of associated adverse events would increase as the dose of cells or density of cell suspension increases. In our recent Phase 1 trial of intravenous hAECs in acute stroke patients, 5 of 8 patients required supplemental oxygen transiently during cell infusion (Phan et al., 2023). However, it is difficult to adjudicate such a risk, as all efforts are made to avoid hypoxia during the infusion process.

The findings of this study firstly suggest that, in our filament-induced model of cerebral ischemia–reperfusion in mice, intravenous tPA can exacerbate early infarct development in association with intracerebral bleeding which may lead to increased 24 h post-stroke mortality. Secondly, intravenous administration of hAECs in combination with tPA can mitigate against infarct development, intracerebral bleeding and 24 h mortality. We conclude that hAECs therapy provides post-stroke cerebroprotection in tPA-treated mice.

## Data availability statement

The raw data supporting the conclusions of this article will be made available by the authors, without undue reservation.

## Ethics statement

The animal study was reviewed and approved by La Trobe University Animal Ethics Committee (AEC 17–79). The collection of amnion epithelial cells was approved by the Monash Health Human Research Ethics Committee. Placenta donors provided their written informed consent.

## Author contributions

CS, HK, SZ, and LB-A designed the experimental protocol and were major contributors in the writing of the manuscript. RL and SC collected cells for experimental administration. LB-A, HK, and SZ performed the animal experimentation and analyses of infarct size, and immunohistochemical markers. TP, HM, GD, and RL contributed to design and interpretation of the data and writing of the manuscript. All authors contributed to the article and approved the submitted version.

## Funding

This study was supported by a donation from the Beluga Foundation and Ideas Grants from the National Health and Medical Research Council of Australia (GNT1163282 and GNT2003156).

## Conflict of interest

The authors declare that the research was conducted in the absence of any commercial or financial relationships that could be construed as a potential conflict of interest.

## Publisher’s note

All claims expressed in this article are solely those of the authors and do not necessarily represent those of their affiliated organizations, or those of the publisher, the editors and the reviewers. Any product that may be evaluated in this article, or claim that may be made by its manufacturer, is not guaranteed or endorsed by the publisher.
