# Supplementary material for: Protection against brain injury after ischemic stroke by intravenous human amnion epithelial cells in combination with tissue plasminogen activator
Source: Front Neurosci. 2023 Jun 16;17:1157236. doi: 10.3389/fnins.2023.1157236 (PMC10311557; doi:10.3389/fnins.2023.1157236)
Supplement: Supplementary file 1 [file Image_1.pdf]

SUPPLEMENTARY MATERIAL

Protection against brain injury after ischemic stroke by intravenous human amnion epithelial cells in combination with tissue plasminogen activator

Liz J Barreto-Arce[1*]; Hyun Ah Kim[1*], PhD; Siow Teng Chan[2,3] PhD, Rebecca Lim[2,3] PhD, Grant R Drummond[1], PhD; Henry Ma[4], MD, PhD; Thanh G Phan[4], MD, PhD; Christopher G Sobey[1+], PhD; and Shenpeng R Zhang[1*], PhD

[1]Department of Microbiology, Anatomy, Physiology and Pharmacology and Centre for Cardiovascular Biology and Disease Research, School of Agriculture, Biomedicine and Environment, La Trobe University, Bundoora, Victoria, Australia.

[2]The Ritchie Centre, Hudson Institute of Medical Research, Clayton, VIC, 3168, Australia.

[3]Department of Obstetrics and Gynaecology, Monash University, Clayton, VIC, 3168, Australia.

[4]Clinical Trials, Imaging and Informatics (CTI) Division, Stroke & Ageing Research (STARC), Department of Medicine, School of Clinical Sciences at Monash Health, Monash University, Clayton, Victoria, Australia.

*Equal contribution; [+]Author for correspondence

Key words: ischemic stroke; neuroprotection; thrombolytic; stem cells; MCAO

Correspondence: Christopher G Sobey, PhD, Department of Microbiology, Anatomy, Physiology and Pharmacology and Centre for Cardiovascular Biology and Disease Research, School of Agriculture, Biomedicine and Environment, La Trobe University, Bundoora, Victoria 3086, Australia.

E-mail c.sobey@latrobe.edu.au

Phone: +61 3 94791316

Twitter: @LaTrobe_CVR, @SRichardZhang, @ChrisSobey4

Supplementary data

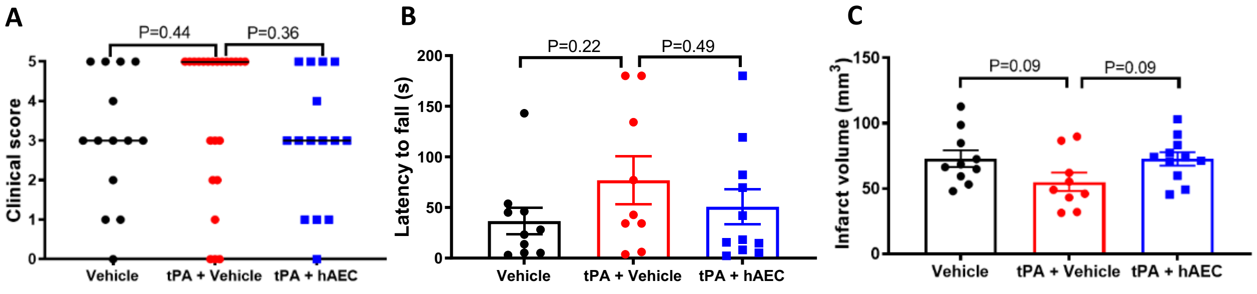

Suppl Figure S1. Functional outcome and infarct volume of mice at 24 h post-stroke. Mice subjected to 1 h transient middle cerebral artery occlusion and tPA-treated at the time of reperfusion were treated with vehicle or hAECs at 1.5 h post-stroke. Functional outcome was evaluated by **(A)** clinical score (n=14-23 per group), **(B)** latency to fall on hanging grip test (n=9-11 per group), and **(C)** infarct volume (n=9-11 per group). Data are presented as mean ± SEM, except for clinical score presented as median. **(A)** Clinical score was compared using a Kruskal-Wallis test. (**B** and **C**) One-way ANOVA test with Dunnett's post hoc test.

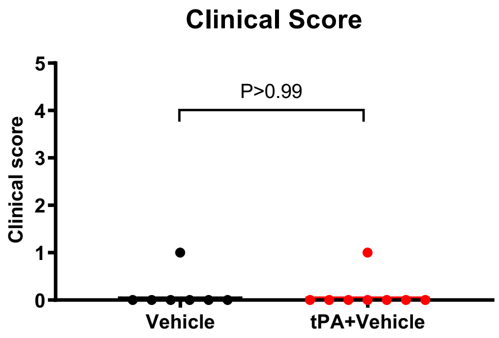

Suppl Figure S2: Clinical score of sham mice at 24 h following vehicle or tPA+vehicle treatment (n=7-8 per group). Data are presented as median and compared using a Mann-Whitney test.

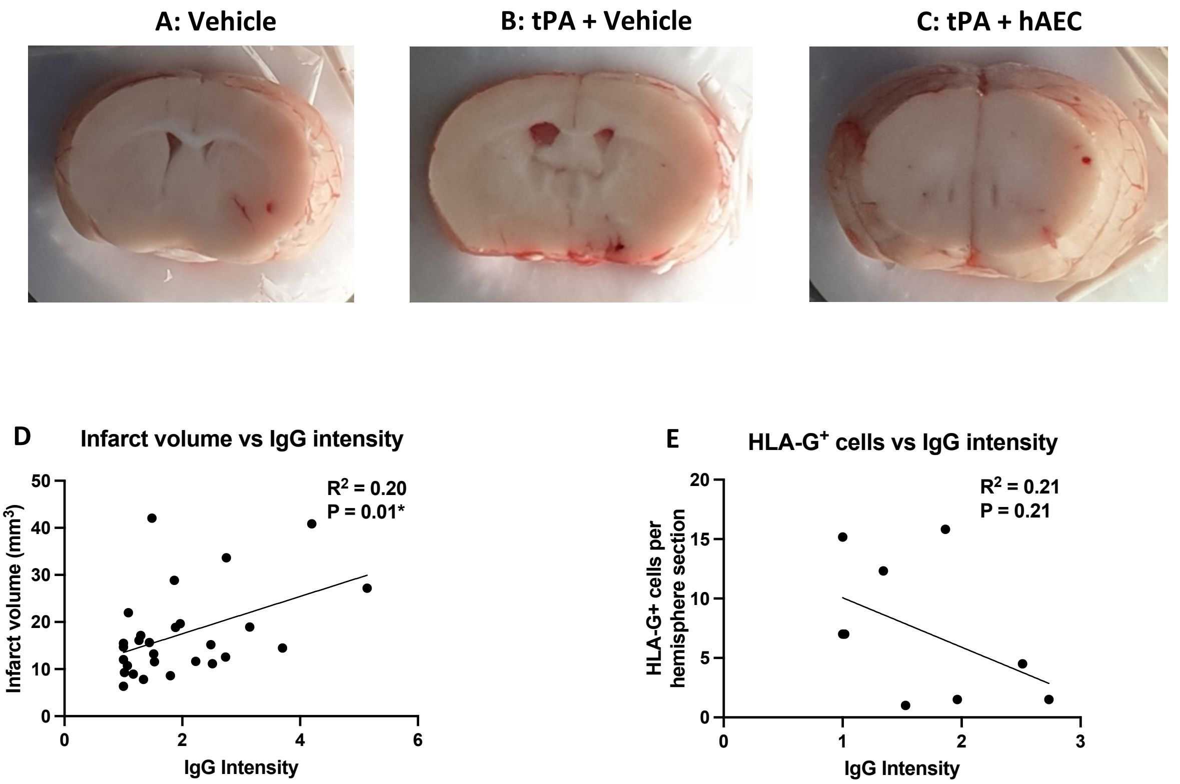

Suppl Figure S3: A-C. Representative images of intracerebral hemorrhage in the ipsilateral hemisphere at 6 h post-stroke. Arrows indicate hemorrhages. **D-E.** Correlation between IgG staining and infarct volume in all mice **(D)**, or between IgG intensity and HLA-G^+ (hAEC) cells in tPA+hAEC-treated mice **(E)**. Coefficient of determination (R^2) and p values are indicated in each graph.

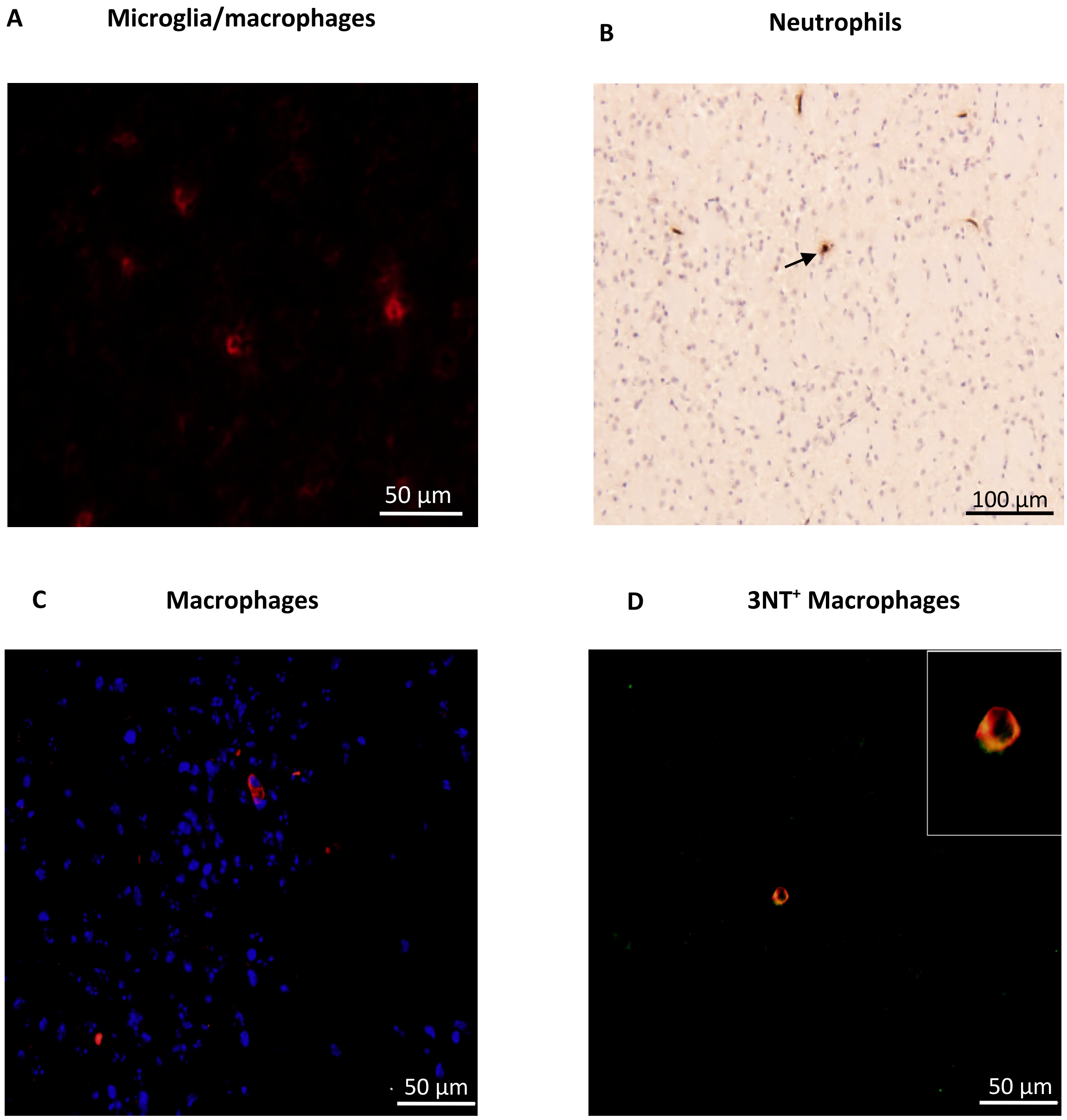

Suppl Figure S4: Representative images of **(A)** microglia/macrophages (Iba-1^{+} cells), **(B)** neutrophils (MPO^{+} cells), **(C)** macrophages (F4/80^{+} cells), nuclei are identified by DAPI counterstain (blue) and **(D)** 3-nitrotyrosine (3NT)$^{+}$ F4/80^{+} cells in the ipsilateral hemisphere at 6 h post-stroke. Objectives: 40X for A, C, D and 20X for B.

END OF DOCUMENT
